# Closed-Loop Gastric Outlet Obstruction Secondary to Duodenal Ulcer in a Patient With Esophageal Stricture

**DOI:** 10.7759/cureus.36507

**Published:** 2023-03-22

**Authors:** Jashandeep Kaur, Georgianna Stoukides, Michael Amaturo

**Affiliations:** 1 Medicine, New York Institute of Technology College of Osteopathic Medicine, Old Westbury, USA; 2 General Surgery, Jamaica Hospital Medical Center, New York, USA

**Keywords:** gastric outlet obstruction, exploratory laparotomy, esophageal stricture, duodenal ulcer disease, zollinger-ellison syndrome

## Abstract

Closed-loop gastric outlet obstruction (GOO) is a rare complication that results from a mechanical obstruction in the pylorus or duodenum. In the early 1990s, the common cause of GOO was peptic ulcer disease, accounting for 5% to 10% of hospital admissions. Peptic ulcer disease is the disruption of the mucosal integrity in the stomach and duodenum and can be categorized into gastric ulcers and duodenal ulcers. With the treatment for *Helicobacter pylori* and the increased use of proton pump inhibitors (PPI), GOO now occurs in fewer than 5% of patients with duodenal ulcer disease and even less in those with gastric ulcer disease. Although the morbidity of duodenal ulcers has been declining in recent years, the incidence of post-bulbar duodenal ulcer (PBDU) remains at a constant 9.33%, primarily due to diagnostic and therapeutic difficulties. Additionally, fewer than 5% of obstructing duodenal ulcers are caused by PBDU, and even fewer are located in the second or third portions of the duodenum. Ulcers located in the distal part of the duodenum raise concern for syndromes associated with hypersecretion of acid, including Zollinger-Ellison syndrome (ZES). The ZES is rare, accounting only for 0.1% of all duodenal ulcers. Here, we present a case where a patient with esophageal stricture developed a rare case of closed-loop GOO secondary to a duodenal ulcer. The patient, initially treated for esophageal perforation, developed an esophageal stricture. The patient was being worked up for ZES and multiple endocrine neoplasia link type 1 (MEN1) syndrome due to his concerning laboratory findings and rare clinical presentation.

## Introduction

Closed-loop gastric outlet obstruction (GOO) is an uncommon complication resulting from a mechanical obstruction in the pylorus or duodenum [1]. Established causes of GOO include peptic ulcer disease, corrosive ingestion, pyloric stenosis, inflammatory diseases, and malignancy [2]. In the early 1990s, GOO was commonly caused by peptic ulcer disease; however, with increased treatment of bacterial causes such as *Helicobacter* *pylori* and the use of medications that decrease gastric acid secretion, GOO is less commonly caused by gastric and duodenal ulcers. In fact, less than 5% of patients with complicated duodenal ulcers develop GOO [2]. Duodenal ulcers occur due to the disruption of mucosal integrity and are included in the broader category of peptic ulcer disease [3]. Duodenal ulcers present commonly in the duodenal bulb area, whereas 5% to 10% of duodenal ulcers occur in the post-bulbar region and, even more rarely, in the third portion of the duodenum [4,5]. Clinical manifestations of post-bulbar duodenal ulcer (PBDU) are still not well established, making diagnosing and treatment difficult [5].

Patients with GOO can present with abdominal pain, nausea, weight loss, melena, and hematemesis [5]. However, many conditions overlap with these symptoms, so a precise diagnosis and treatment are needed to address duodenal ulcers before complications like GOO occur. Additionally, ulcers in the distal two-thirds of the duodenum raise concern for syndromes associated with hypersecretion of acid, such as Zollinger-Ellison syndrome (ZES). This syndrome is characterized as a group of symptoms occurring due to an increase in gastric acid secretion, including peptic ulcer disease, gastroesophageal reflux disease (GERD), and gastrin-secreting tumor of the duodenum [6]. High clinical suspicion is required to diagnose ZES as symptoms are often nonspecific and overlap with other diseases [7]. Although hypergastrinemia is considered the hallmark of ZES, it can also occur with proton pump inhibitor (PPI) therapy and GOO, and thus is nonspecific [8]. Patients can develop ZES due to a genetic syndrome called multiple endocrine neoplasia type 1 (MEN1). Th MEN1 is a collection of disorders present in patients with a mutation in the MEN1 gene, leading to overly active endocrine glands [9]. The group of disorders includes hyperparathyroidism, pancreatic endocrine neoplasm, and pituitary neoplasm. Multiple endocrine neoplasia type 1 has been linked to ZES, as approximately 25% of patients with MEN1 also have ZES [10]. Patients with MEN1 and ZES may present with esophagitis, dysphagia, and heartburn, and are three times more likely to have esophageal strictures and duodenal ulcers. However, ZES is rare and only accounts for 0.1% of all duodenal ulcer diseases [11]. 

The patient presented in this case report suffered from GERD-related esophagitis and stricture for months. He was diagnosed with MEN1-related hyperparathyroidism. He had significant laboratory findings of hypergastrinemia and hypercalcemia, and presented with severe abdominal pain and a CT finding of pyloric and duodenal thickening and ulcerations. The duodenal ulcerations caused upper gastrointestinal (UGI) bleeding that resulted in hemodynamic instability and caused a rare case of closed-loop GOO.

## Case presentation

**Figure 1:**
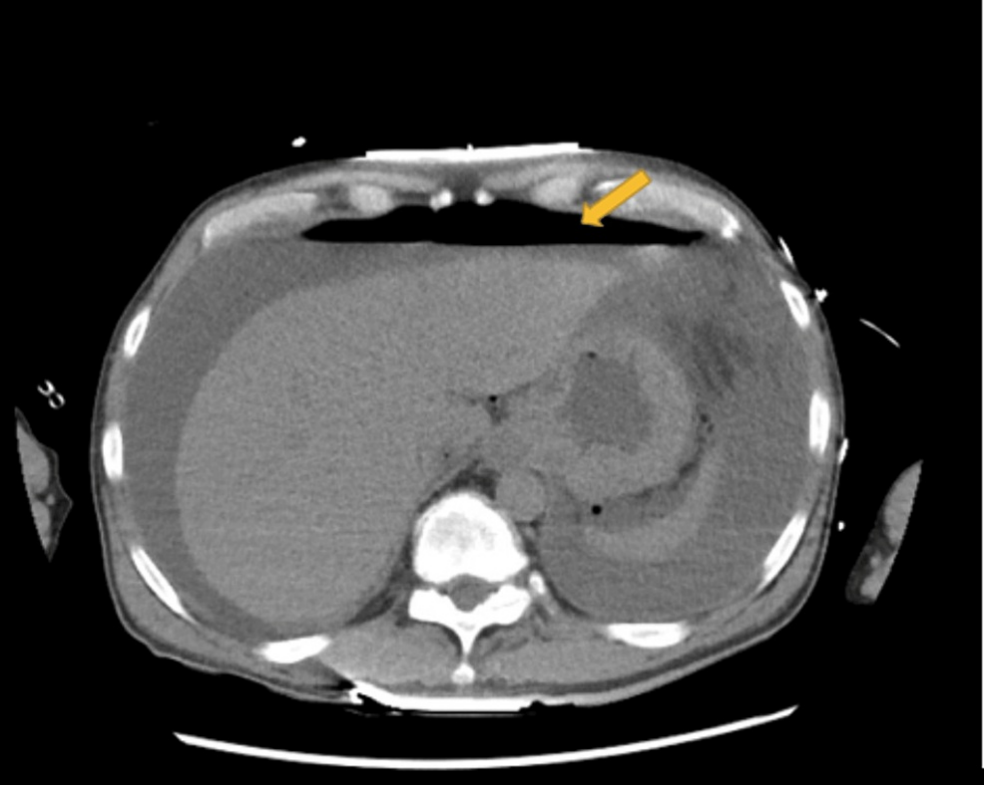
CT scan of the abdomen and pelvis showing pneumoperitoneum (yellow arrow)

**Figure 2:**
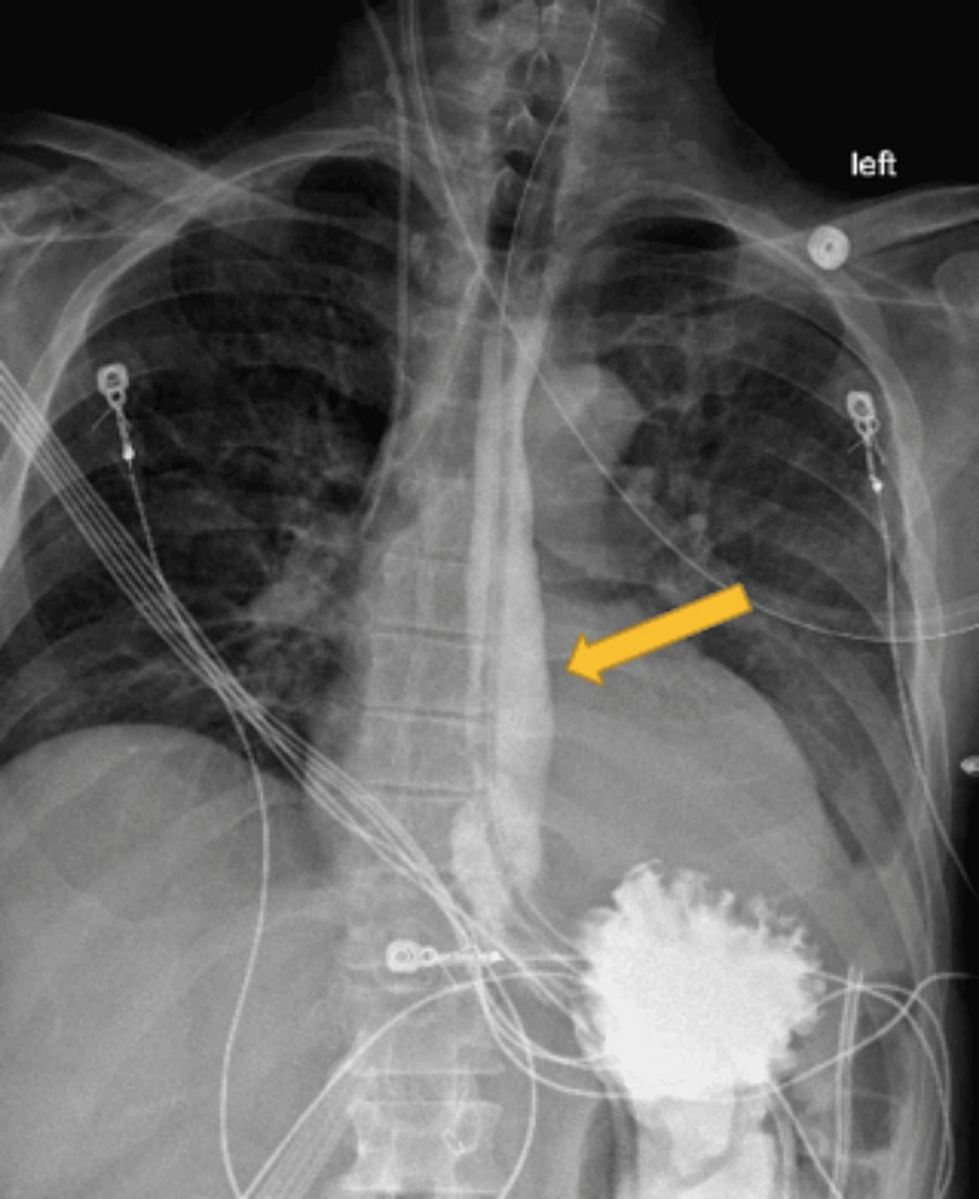
Barium swallow two weeks after esophageal perforation repair. At this point, the esophagus (yellow arrow) is of normal caliber.

A 55-year-old man with a medical history of GERD esophagitis, esophageal stricture, and MEN1-related hyperparathyroidism was admitted to the hospital for chest pain and esophagitis. Three days post-admission, the patient decompensated and had episodes of hypotension with systolic blood pressure in the 60s. A CT scan of the abdomen and pelvis revealed pneumoperitoneum and fluid of unclear source (Figure 1). Surgery was consulted and the patient consented to an emergent exploratory laparotomy. During the exploratory laparotomy, a 5 cm perforated distal esophagus was located. An esophagogastroduodenoscopy (EGD) was performed to confirm the location was in the distal esophagus and the stomach was normal. Primary repair with omental plication was then performed. The patient was admitted to the surgical intensive care unit (SICU) post-surgery. Postoperative UGI studies were obtained that showed no evidence of leakage (Figure 2). The gastrointestinal examination was normal; however, the patient had emergent left aortoiliac, femoral-popliteal thromboembolectomy, and compartment fasciotomy after a physical exam revealed a cold lower limb and absent pedal pulses. Once the patient was stabilized and able to tolerate diet, he was discharged. 

**Figure 3:**
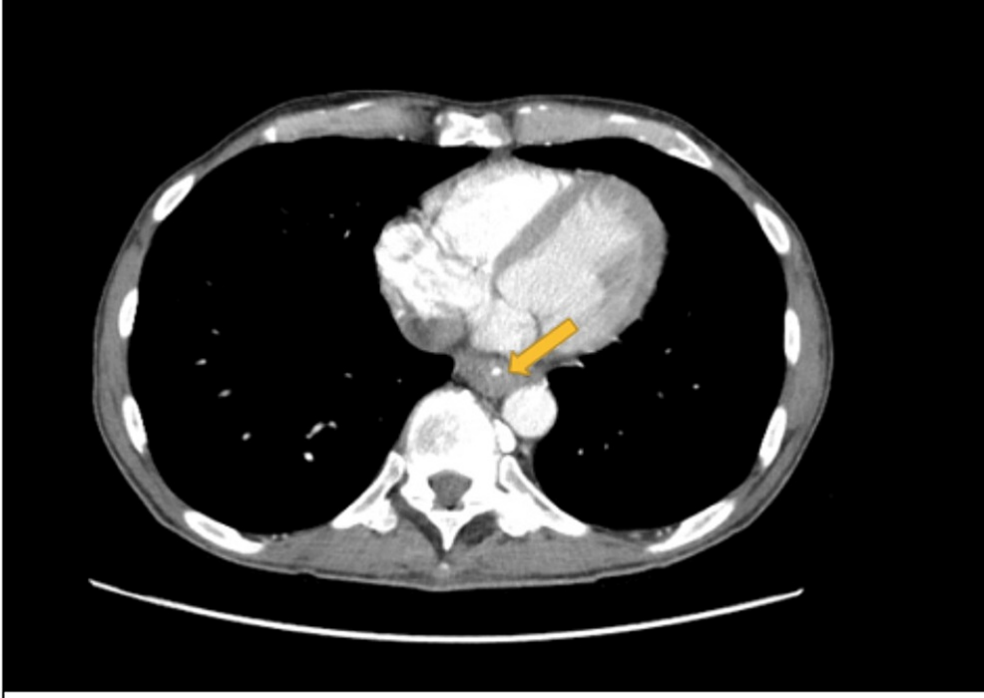
CT chest with contrast showing esophageal thickening (yellow arrow)

**Figure 4:**
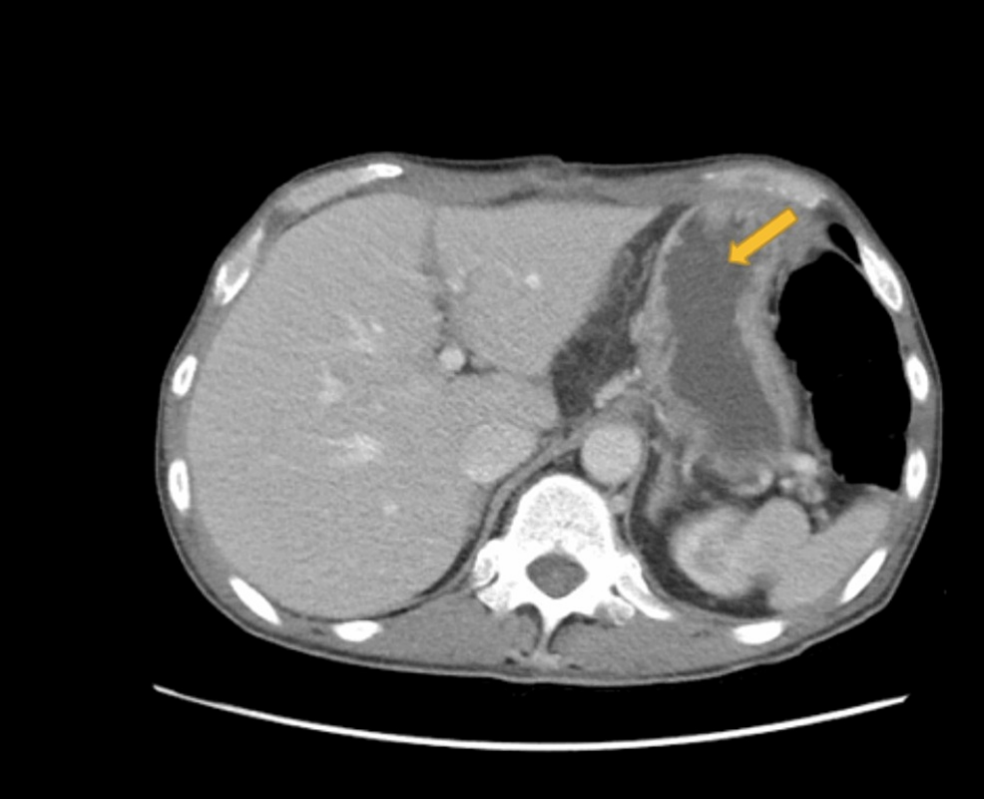
CT of abdomen and pelvis with contrast showing thickening involving the descending duodenum (yellow arrow)

**Figure 5:**
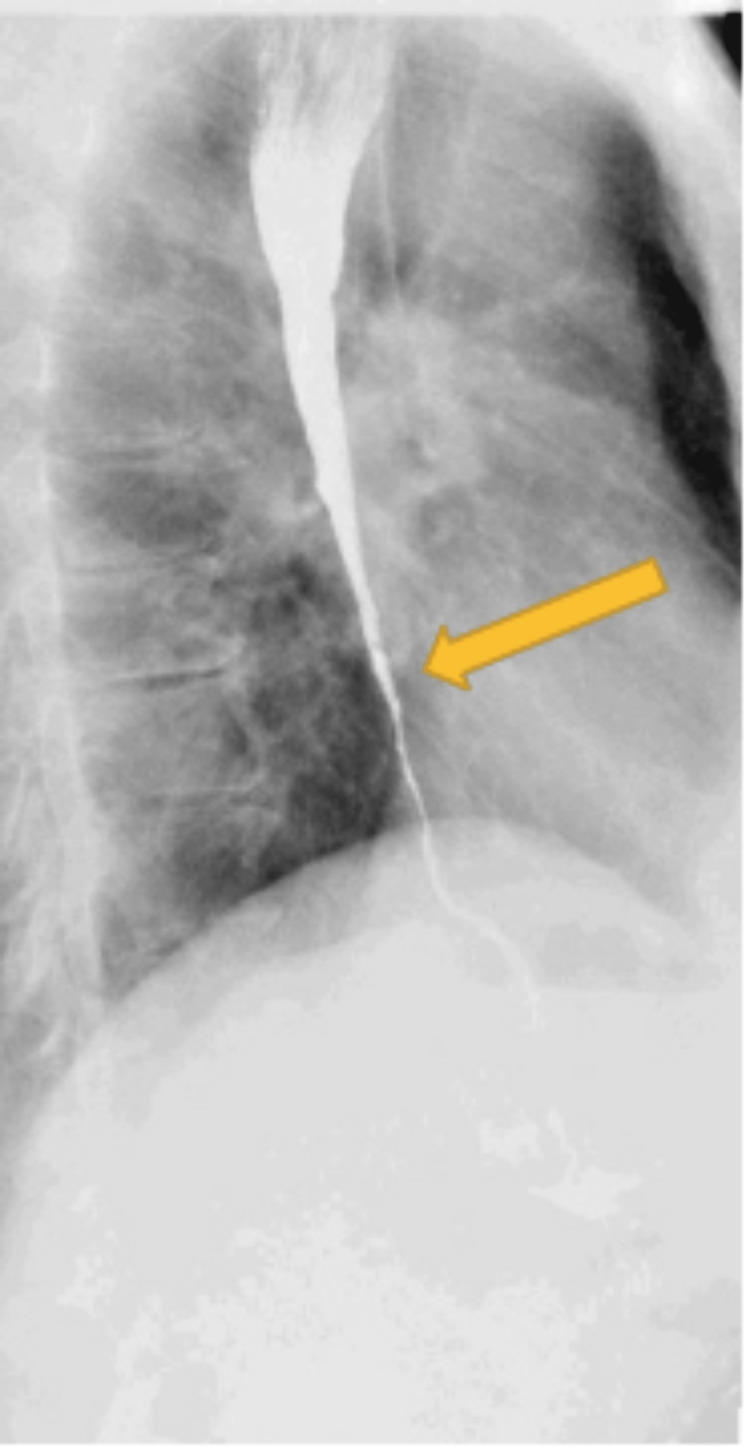
Barium swallow image six weeks after initial presentation showing severe stenosis of the distal esophagus (yellow arrow)

Two days after being discharged from the hospital, the patient returned to the ED with complaints of abdominal pain, emesis, dysphagia, lack of flatulence, and bowel movement. The patient was admitted to the surgery department with a temperature of 98.1^o^F, blood pressure of 100/62 mmHg, pulse of 120 beats/min, respiratory rate of 17 breaths/min, and BMI of 18.56kg/m^2^. The complete blood count (CBC) showed an elevated white blood cell count of 23, and an alkaline phosphatase of 188. Creatinine, blood urea nitrogen (BUN), and bicarbonate were normal. A CT of the chest with contrast revealed esophageal mural thickening and edema prominent in the distal half of the esophagus (Figure 3). A CT of the abdomen and pelvis with contrast showed mild gastric and pyloric mural thickening involving descending duodenum and proximal third portion of the duodenum (Figure 4). The patient was admitted for observation and administered a high dose of PPI pantoprazole IV 40mg and resuscitation fluids. Gastroenterology was then consulted and recommended a barium swallow and UGI series. Impressions from the tests included long segment narrowing of the mid and distal esophagus (Figure 5).

**Figure 6:**
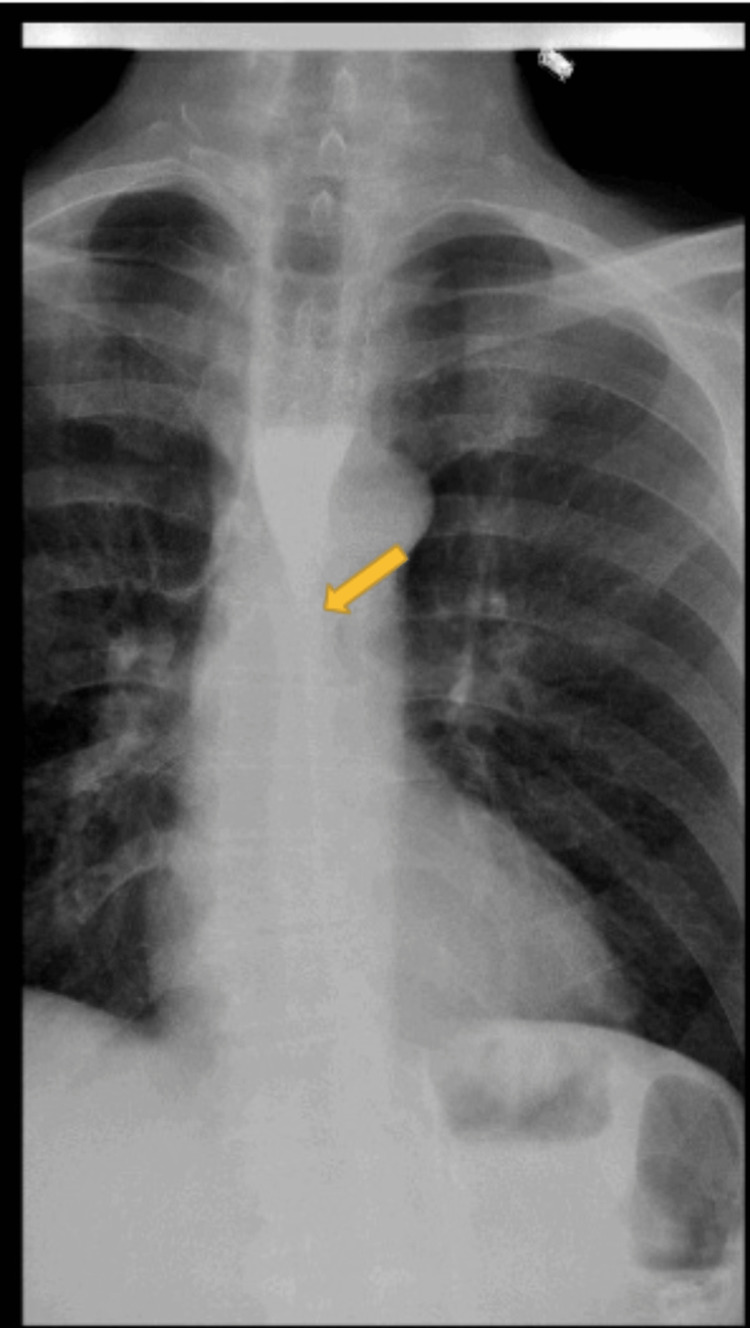
Fluoroscopy esophagram showing luminal narrowing of mid to distal esophagus (yellow arrow)

An EGD with flexible esophagoscopy and esophageal dilation revealed esophageal stricture 30cm from the lips. Esophagitis grade 4 was noted distal to the stricture and only a 5 mm Savary dilator was able to pass through the stricture. A fluoroscopy esophagram was also performed, showing luminal narrowing of the mid to distal esophagus compatible with esophageal stricture (Figure 6). The plan at this time was to discharge the patient with a jejunostomy tube (J-tube) to a skilled nursing facility for the management of tube feedings and follow up with an outpatient gastroenterologist for serial dilations of the esophagus. 

**Figure 7:**
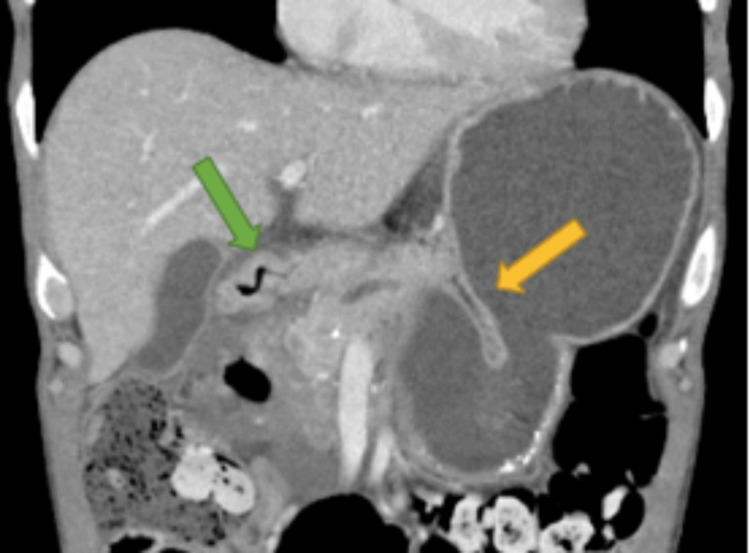
CT abdomen and pelvis with IV contrast showing severe stenosis at the second portion of the duodenum with ulcer (green arrow), and a gastric closed loop obstruction (yellow arrow)

Seven weeks later, the patient returned to the ED feeling unwell. A CT of the abdomen and pelvis revealed severe inflammation of the duodenum with probable ulceration in the second part distally likely causing GOO (Figure 7). The patient was suggested to be evaluated for ZES related to the reported MEN1 diagnosis. Interventional radiology was consulted to place a guided percutaneous gastrostomy catheter (G-tube) to allow for the decompression of the stomach. The patient continued to be monitored with the plan of being transferred to a tertiary care center; however, on hospital day 9 the patient presented with melanotic stool and signs of hemorrhagic shock, suggestive of a gastrointestinal bleed. Radiology was consulted for an angiogram of the celiac axis and gastroduodenal artery embolization. On hospital day 10, the patient presented as acutely distressed with a pulse of 150 beats/min and blood pressure of 70/50mmHg. Exploratory laparotomy revealed an ulcer at the second and third portions of the distal duodenum, distal to the ampulla of Vater, and near the medial side of the head of the pancreas. Adhesions between the gallbladder and duodenum and between the transverse colon and duodenum were lysed, revealing an anterior perforation that was being sealed by these adhesions. The duodenal mucosa was imbricated around the ulcer. The omental patch from the hepatic flexure of the transverse colon was also imbricated. The patient remained in the SICU during post-operation and was then transferred to a tertiary care center for further care.

## Discussion

Gastric outlet obstruction results from a mechanical obstruction in the distal stomach, pylorus, or duodenum causing nausea, vomiting, early satiety, weight loss, and abdominal pain. Benign causes of GOO include ulcers, non-steroidal anti-inflammatory drugs (NSAIDs), pancreatitis, and percutaneous endoscopic gastrostomy tube migration. Patients with GOO associated with ulcers generally have a long-standing history of symptoms including dyspepsia and weight loss. Gastric outlet obstruction secondary to ulcers occurs because of tissue inflammation and edema in acute cases, and scarring and fibrosis with strictures in chronic cases [12]. Our patient presented with abdominal pain, nausea, and weight loss.

Clinical manifestations of GOO have been extensively documented. During a physical examination, there are signs of gastric obstruction that can present as cachexia, malnutrition, succussion splash, or palpable abdominal mass. Further studies may show signs of electrolyte imbalance secondary to nausea and vomiting. Plain radiography can be used to diagnose obstruction as it may reveal an enlarged gastric bubble, and contrast may be added to determine complete versus partial obstruction. A CT or MRI may also be used to diagnose GOO, as it may show gastric distention due to a gastric, pyloric, duodenal, or pancreatic mass causing the obstruction [12]. An upper endoscopy can also establish the diagnosis and cause of GOO [12]. For our patient, CT imaging showed the esophagus with mural thickening and infiltration of adjacent fat, duodenal thickening with duodenal ulcer, and probable GOO.

**Figure 8:**
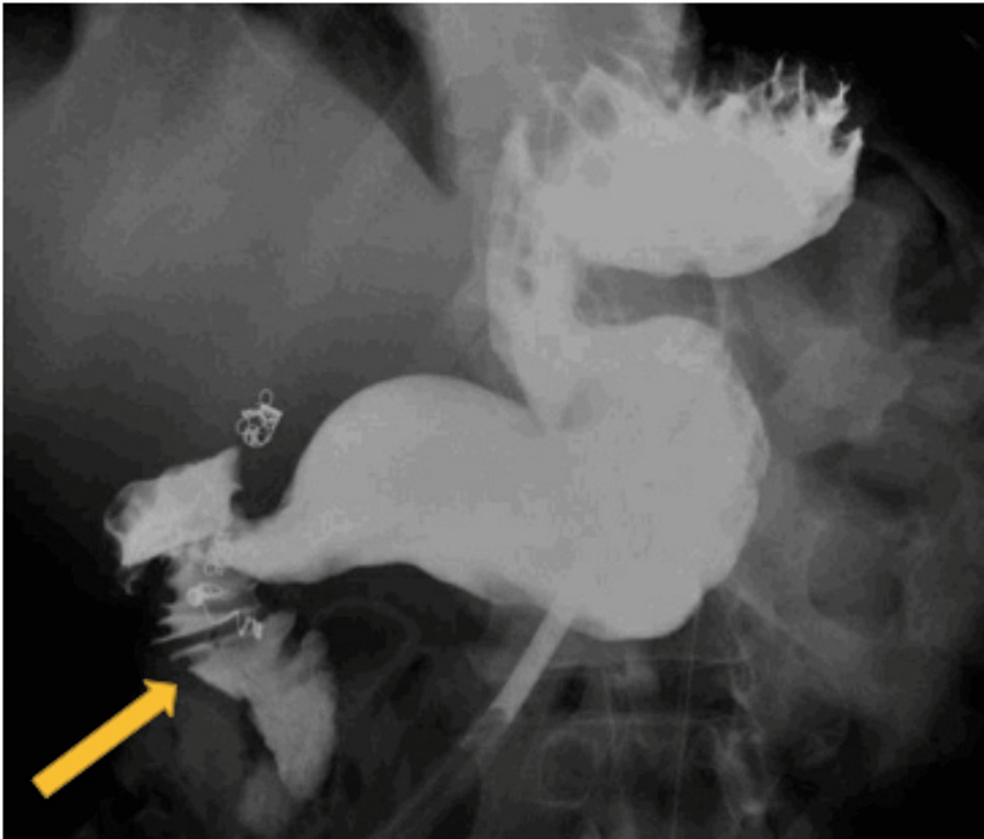
Upper gastrointestinal series with enteral contrast via the gastrostomy tube showing the contrast past the second portion of the duodenum (yellow arrow)

Patients with signs and symptoms of GOO should be given nothing by mouth (NPO), PPI to decrease gastric secretions, and intravenous fluids to replenish volume depletion [12]. Management of our patient's GOO included endoscopic dilation; however, due to the complexity of the esophageal narrowing, endoscopic dilation could not be performed and was to be done at a tertiary care center. Surgery is indicated when medical management fails. During this patient’s admission, he had a G-tube placed to allow for decompression of the stomach after CT showed inflammation in the duodenum and probable GOO causing a closed-loop obstruction (Figure 8). However, on the 10th-day post-admission, he had melanotic stool and signs of UGI bleeding. Interventional radiology was consulted to embolize the gastroduodenal artery. This failed, as the patient presented with hemodynamic instability and had to proceed to an exploratory laparotomy. During this operation, an ulcer was located in the distal duodenum, distal to the ampulla of Vater, near the medial side of the head of the pancreas. This ulcer was the cause of bleeding from the transverse colon mesentery. The anterior perforation was sealed by the gallbladder and transverse colon adhesions to the duodenum. This is a rare and unusual location for duodenal ulcers to appear, as they are most commonly present in the pyloric area. The duodenal ulcer was managed during surgery by oversewing the ulcer and using an omental patch.

This patient had a high suspicion for combined ZES and MEN1 syndrome as he previously presented with concerning relevant symptoms and had an elevated gastrin level of 426 pg/ml while on high-dose PPIs. However, hypergastrinemia is not limited to ZES and MEN1 syndromes as it can also be seen in patients undergoing PPI therapy, hypercalcemia, or GOO [9]. The ZES is a group of symptoms caused by increased gastric acid secretion due to gastrin-secreting tumors, and gastrinomas [7,11]. Our patient presented with all of these findings as his past medical history included GERD and hypercalcemia. To localize gastrinomas, CT scans of the abdomen, scintigraphy, endoscopic ultrasonography, and PET scans are used [9]. Although gastrinomas were not visualized or attempted to be visualized in the imaging for this patient, a careful history and physical examination should help discern ZES from other causes. Additionally, gastrinomas are typically found in the duodenal area, so meticulous observation in this area to locate the neuroendocrine tumor should be considered especially during surgery involving this area. Surgery is a curative treatment for gastrinomas in absence of metastases, and duodenal gastrinomas result in duodenectomy [9]. For this patient, gastrinomas were not observed during the exploratory laparoscopy as there were concerns for the patient’s hemodynamic stability and excessive gastrointestinal bleeding. 

The ZES results in the development of ulcers in 67% of patients, esophageal stricture in 42% of patients, and bleeding in 12% of patients [11]. When ZES is suspected, it is recommended to start the patient on high-dose PPI therapy. Although our patient was being treated for GERD with high-dose PPIs and was being observed for his esophageal stricture, he still developed a duodenal ulcer that led to closed-loop GOO. In the future, to decrease the risk of closed-loop GOO in patients with symptoms of ZES, it may be advised that they undergo observation of gastrinomas in the duodenum and PBDU. From our literature review, this may be the first reported case of a gastric closed-loop obstruction and rare presentation of a duodenal ulcer in the lower thirds of the duodenum [13].

## Conclusions

Duodenal ulcers located in the second and third parts of the duodenum and closed-loop GOO are rare complications. We discussed a case of closed-loop obstruction due to a proximal esophageal stricture and distal GOO. Our patient had a hemodynamically unstable presentation while in the intensive care unit, suspicious for gastrointestinal bleed status-post G-tube placement. An exploratory laparotomy revealed duodenal ulcers that were then repaired by oversewing the omentum. The patient was then transferred to a tertiary care center for an esophagectomy. At the six-month follow-up, the patient continued to suffer from intestinal perforations. At the time of this publication, the patient is in critical condition in the SICU.
